# Generating super-shedders: co-infection increases bacterial load and egg production of a gastrointestinal helminth

**DOI:** 10.1098/rsif.2012.0588

**Published:** 2013-03-06

**Authors:** Sandra Lass, Peter J. Hudson, Juilee Thakar, Jasmina Saric, Eric Harvill, Réka Albert, Sarah E. Perkins

**Affiliations:** 1 Center for Infectious Disease Dynamics, Pennsylvania State University, University Park, PA 16802, USA; 2 Department of Physics, Pennsylvania State University, University Park, PA 16802, USA; 3 Department of Veterinary and Biomedical Sciences, Pennsylvania State University, University Park, PA 16802, USA; 4 Cardiff School of Biosciences, Sir Martin Evans Building, Museum Avenue, Cardiff CF10 3AX, UK; 5 Institute of Pathology, Albert-Ludwigs-University Freiburg, University Medical Center, Breisacher Strasse 115a, Freiburg 79006, Germany; 6 Biomolecular Medicine, Department of Surgery and Cancer, Faculty of Medicine, Imperial College London, Sir Alexander Fleming Building, South Kensington, London SW7 2AZ, UK

**Keywords:** co-infection, super-shedders, bioluminescence imaging, *in vivo* disease dynamics, immune-mediated interactions

## Abstract

Co-infection by multiple parasites is common within individuals. Interactions between co-infecting parasites include resource competition, direct competition and immune-mediated interactions and each are likely to alter the dynamics of single parasites. We posit that co-infection is a driver of variation in parasite establishment and growth, ultimately altering the production of parasite transmission stages. To test this hypothesis, three different treatment groups of laboratory mice were infected with the gastrointestinal helminth *Heligmosomoides polygyrus*, the respiratory bacterial pathogen *Bordetella bronchiseptica* lux^+^ or co-infected with both parasites. To follow co-infection simultaneously, self-bioluminescent bacteria were used to quantify infection *in vivo* and in real-time, while helminth egg production was monitored in real-time using faecal samples. Co-infection resulted in high bacterial loads early in the infection (within the first 5 days) that could cause host mortality. Co-infection also produced helminth ‘super-shedders’; individuals that chronically shed the helminth eggs in larger than average numbers. Our study shows that co-infection may be one of the underlying mechanisms for the often-observed high variance in parasite load and shedding rates, and should thus be taken into consideration for disease management and control. Further, using self-bioluminescent bacterial reporters allowed quantification of the progression of infection within the whole animal of the same individuals at a fine temporal scale (daily) and significantly reduced the number of animals used (by 85%) compared with experiments that do not use *in vivo* techniques. Thus, we present bioluminescent imaging as a novel, non-invasive tool offering great potential to be taken forward into other applications of infectious disease ecology.

## 1. Introduction

A dominant feature of host–parasite interactions is the large variation in infection and infectiousness. Individuals infected with HIV, for example, may either rapidly develop acquired immune deficiency syndrome, or take many years before showing overt symptoms [1]. In a similar manner, individuals exhibit large variation in parasite infection with some remaining chronic while others are rapidly cleared [2]. The significant variation in the establishment and growth of parasites among individuals is such that few individuals are responsible for a large proportion of the transmission events [3,4]. One striking example was illustrated by the severe acute respiratory syndrome epidemic where 103 of the first 201 cases were infected by just five source cases—individuals termed super-spreaders, i.e. those that infect an unusually large number of secondary cases [5–8]. Typically, super-spreaders are those with higher than average contact rates although increased host infectiousness is also implicated in super-spreading events [5,8]. Individuals with the potential to be highly infectious can be referred to as a ‘super-shedders’; individuals that for a period of time yield many more infective stages than most other infected individuals of the same host species [5]. A major challenge in disease biology is to identify some of the mechanisms that may generate super-shedders. One of the drivers of variation in parasite load and host infectiousness is proposed to be underlying secondary infection, or co-infection, the simultaneous infection of an individual with two or more parasitic species [9–11]. We investigate the hypothesis that co-infection alters the likelihood of parasite establishment, growth and shedding of both parasites and may generate super-shedders. Here, we use the term parasite to include both macroparasites (helminths, protozoa) and microparasites (viruses and bacteria).

Co-infection is common across species [12] and so understanding the mechanics of co-infection, i.e. the effect on growth and establishment of one parasite on another is important for host health and effective disease control. When a co-infection consists of helminth and bacteria that infect different organs within an individual the variation in parasite establishment and growth may be expected to be resource or immune-mediated [13–19]. In the case of the latter, a broad antagonism in immune mechanisms in response to the two different types of parasite may be expected to generate differential infection dynamics [20]. Helminths typically induce cytokines associated with a T-helper cell type 2 (Th2) immune response, which simultaneously downregulates T-helper cell type 1 (Th1) cytokines, which are involved in fighting intracellular microparasites [20]. As such, this antagonism may alter co-infection dynamics, and this has been shown to be the case using mathematical models [15], whereas meta-analyses of empirical data have identified key cytokines that may be broadly accountable for shaping co-infection dynamics [13]. It is therefore likely that variation in individual parasite species' establishment, growth and ultimately host infectiousness may be a function of co-infection.

We undertook a detailed longitudinal study of parasite load *in vivo* and in real-time to quantify simultaneously the mechanics of co-infection, i.e. the parasite establishment and growth of the bacterial parasite and establishment and transmission potential of the helminth (as measured by egg production stages) during co-infection, in comparison with single infection scenarios. We examined the establishment and growth of the respiratory bacterium *Bordetella bronchiseptica* lux^+^ (a self-bioluminescent strain), using bioluminescence imaging (BLI). Simultaneously, we monitored the shedding of eggs of the directly transmitted gastrointestinal helminth *Heligmosomoides polygyrus* in laboratory mice*.* Both parasites are well studied immunologically [21–24] and inhabit different physical locations in the host and so are unlikely to directly compete for resources or otherwise, therefore we hypothesize that interactions will likely be immune-mediated.

## 2. Methods

### 2.1. How does co-infection alter parasite establishment, growth and load?

Quantitative counts of self-bioluminescent bacteria were made *in vivo* (the bacterial load), whereas the number of eggs shed by helminths and their development to infective stages (L3 larvae) were used as a proxy measure of helminth infectiousness. Daily measurement of the bacterial load was carried out *in vivo* until equilibrium densities were reached, whereby a persistent nasal infection was observed for more than 5 days in a row (a total of 22 days for bacteria) or no eggs were shed (365 days for helminths). A controlled laboratory setting was used to eliminate confounding factors known to cause variation in parasite dynamics, including environment, nutrition, prior parasite exposure, host genetics, sex and age [25–28]. All mice used were females, aged six to eight weeks caged individually in randomized locations within a single room in an animal facility for the duration of the experiment and provided with food ad libitum.

A total of 40 female BALB/c mice (The Jackson Laboratory, Bar Harbor, ME, USA) were randomly allocated to one of four treatment groups (10 in each group): (i) inoculation with the respiratory pathogen *B. bronchiseptica* lux^+^; (ii) inoculation with the helminth *H. polygyrus*; (iii) simultaneous inoculation with *B. bronchiseptica* lux^+^ and *H. polygyrus*; (iv) a control group that received sham inocula of culture medium. We used *B. bronchiseptica* strain RB50, which had been rendered self-bioluminescent by the chromosomal insertion of a plasmid—pSS4266 to produce *B. bronchiseptica* lux^+^ [29]. The lux operon is driven by the *fha* promoter and is constitutively expressed such that bacteria are self-bioluminescent (i.e. do not require addition of a substrate), and the light emitted was quantified *in vivo* over time using BLI. Bacterial doses were confirmed by plating dilutions and carrying out colony counts prior to inoculation. Mice were intra-nasally inoculated under light anaesthesia (continuous flow 5% isoflurane in oxygen) with a 50 μl droplet of *B. bronchiseptica* lux^+^ in PBS+1 per cent Stainer–Scholte medium (*ca* 10^4^ bacterial cells). The helminth-only treatment group received an intranasal sham-inoculation of 50 μl PBS+1 per cent Stainer–Scholte medium. Mice assigned to a co-infection or helminth-only treatment were simultaneously inoculated with 180±30 *H. polygyrus* infective L3 larvae in 20 μl distilled water, administered via oral gavage. The mean number of larvae in each inoculum was estimated from 10 direct counts of larvae in 20 μl of water prior to gavaging. The bacteria only and control treatment groups received a sham-inoculation of 20 μl distilled water, administered via oral gavage. Inoculation of animals was carried out in a random order on day zero of the experiment.

### 2.2. Bioluminescence imaging

Mice were placed in groups of up to three, within their treatment group inside an IVIS 50 (Caliper Life Sciences, Hopkinton, MA, USA). Mice were anaesthetized using a continuous flow of 5 per cent isoflurane mixed with oxygen for 5 min to allow acquisition of an image quantifying the light emission *in vivo* from *B. bronchiseptica* lux^+^. Bacterial load measurements were obtained from three regions of the mouse: (i) whole body, within a square of standard size 3.0 cm width and 5.0 cm height, (ii) head, including nose and trachea using an elliptical region of size 2.3 cm width and 2.0 cm height, and (iii) lungs using a standardized square region of 3.0 cm width and 3.0 cm height. Mice were imaged at approximately the same time of day, from day zero (just prior to inoculation) until day 22. An image was acquired over a 5 minute period and the software Living Image (v. 2.6.1, Xenogen Corporation, Almeda, TX, USA) was used to convert the photons emitted from *B. bronchiseptica* lux^+^ within the host into relative light units (RLUs). The spatial location of infection in the mouse was overlaid on a photographic image using a pseudo-colour to quantify the RLUs. Previous studies have shown the light output (RLUs) from self-bioluminescent bacteria to correlate positively with viable counts of bacteria *in vivo* [30–33], thereby giving a real-time quantification of bacterial load. To validate the relationship between *B. bronchiseptica in vivo* and *in vitro*, comparisons were made between viable counts of bacteria from a parallel experiment where animals were sacrificed at set time points (see §2.1.3.) and related to the RLUs *in vivo*.

To determine whether the time course of bacterial load differed between single and co-infected treatment groups we used the model described by Fenton *et al.* [34], where a generalized linear mixed model (GLMM) using ASReml v. 2.0 was used to determine differences in the number of faecal egg counts of multiple individuals over time. Mixed models allow the user to control for multiple variables at the same time, including both random and fixed effects. One advantage to ASReml is that spline terms are included in the random model where only 1 d.f. is used. Here, we fit this spline to the time course of infection in our data, allowing nonlinear relationships between variables to be modelled. We also included host identification as a random term to control for pseudo-replication (i.e. autocorrelation errors). For the fixed model, we fitted a spline to the bacterial load, over time, of the single and co-infected groups and used this as the response variable with treatment group (single or co-infected) as the explanatory variable. We first log-transformed the data and then carried out a GLMM analysis using ASReml in software R [35]. To assess whether co-infection altered host mortality, Cox proportional hazards were used to determine how survivorship differed between the treatment groups [35].

### 2.3. How does co-infection alter helminth egg shedding?

Helminth eggs were counted from host faecal samples, collected at approximately the same time of the day, from day 5 post-inoculation, i.e. just before the time point when helminth larvae moult into adults [36]. We monitored helminth egg shedding every 3 days until day 44, thereafter weekly sampling occurred until no eggs were found in an individual for three consecutive time points (last time point collected was day 365). Prior to collecting faeces for egg counts, mouse cages were cleaned at 16.00±2 h, a subsample of faeces was collected approximately 18 h later, and helminth eggs quantified using standard McMaster techniques. To determine whether co-infected mice shed significantly more helminth eggs than single-infected mice, over time, we carried out a GLMM with random terms using ASReml in R (after [34] and [35]). A unique identification number for each animal was included in the model as a random term to remove the variation caused by individuals in order to gain insights into any underlying relationships in the fixed model. For the fixed model, we fitted a spline to the parasitic load, over time, of the single and co-infected groups and used this as the response variable with treatment group as the explanatory variable. To quantify whether co-infection generated super-shedders, we used the distribution of eggs shed from the helminth-only infected animals as a baseline distribution. We defined super-shedders as individuals (from the co-infected treatment group) that were 2 s.d.s above the mean of single-infected individuals.

To determine that the number of helminth, eggs shed were a good proxy for transmission potential of the helminth we cultured helminth eggs from faecal samples to their infectious L3 larval stage. After 10 days of culture at 20°C to ensure the inclusion of early and late hatching larvae, we quantified the L3 stages produced using a ‘whites trap’ [37,38] using faeces from the single (*n* = 9) and co-infected animals (*n* = 7) on day 21 post-inoculation. To assess whether differences existed between co-infected and single-infected groups in the viability of eggs maturing to the L3 infectious stage, we carried out a GLM with normally distributed errors with treatment as the explanatory variable (i.e. single or co-infected) and the number of L3 larvae per gram of faeces as a response variable [35].

To validate the *in vivo* techniques; that helminth infections had established and that the RLUs of the bacteria were positively associated with the viable counts of bacteria in an individual, we carried out destructive sampling in parallel with the *in vivo* experiment. An additional 72 female mice (four treatments × six time points × three replicates) were infected and kept under the same conditions as the *in vivo* treatment groups (*H. polygyrus* only, *B. Bordetella* lux^+^ only, co-infection and control). Three animals per treatment group were euthanized at days 0, 3, 6, 12, 24 and 48 post-inoculation. Animals were sacrificed via CO_2_ inhalation and the lungs, trachea, nasal cavity and gastrointestinal tract were removed. The gastrointestinal tract was immediately dissected at 10× magnification in Hank's balanced salt solution and adult *H. polygyrus* counted to determine whether the number of adult helminths that had established in mice differed between single and co-infected individuals. We analysed these data using a GLM with negative binomial errors with treatment and time points as fixed effects and the number of adult helminths as response variable. To assess how the number of helminth eggs shed correspond to helminth infection intensity, we also collected faecal samples from these animals prior to euthanasia. We calculated the number of eggs per gram (EPG) faeces per helminth and determined whether the number of eggs per helminth differed between treatments using a GLM with negative binomial errors with treatment and time of sampling as fixed effects [35].

To relate viable counts of bacteria to the RLUs measured *in vivo* and to determine the viability of the *B. bronchiseptica* lux^+^ the lungs, nose and trachea were homogenized in PBS and serial dilutions used to count colonies. *Bordetella bronchiseptica* lux^+^ was plated onto growth medium using standard techniques [21], and the colony-forming units (CFUs) were counted after incubation at 37°C for 2 days [21].The relationship between the CFUs and the lux measurements *in vivo* was assessed using correlation analysis.

## 3. Results

### 3.1. How does co-infection alter bacterial establishment, growth and load?

**Figure 1.**
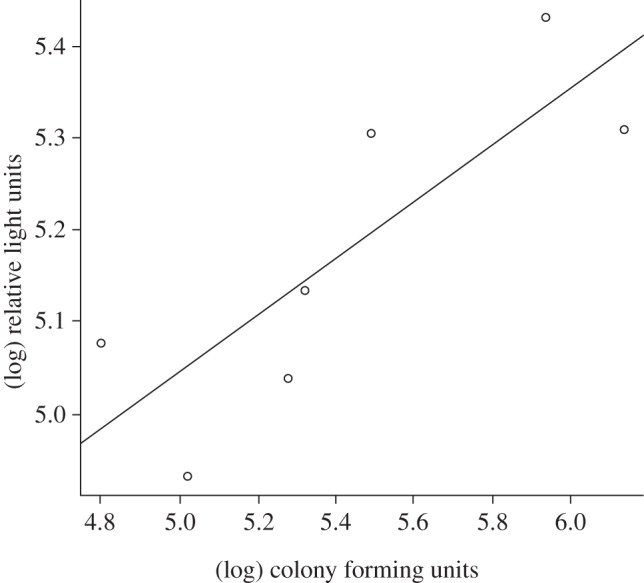
Correlation between light output (measured in relative light units) and colony-forming units in hosts infected with self-bioluminescent *Bordetella bronchiseptica* lux^+^.

The efficiency of the reporter bacteria was assessed first, and it was determined that light output (RLUs) from self-bioluminescent bacteria measured *in vivo* was positively correlated (*r* = 0.82; *p* < 0.05) with the CFUs of bacteria from animals that were sacrificed at days 3, 6, 12 and 24 (figure 1).

**Figure 2.**
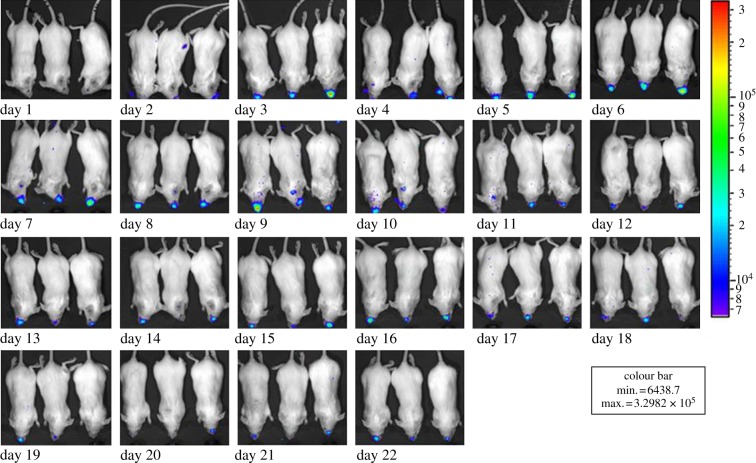
Bacterial load over time of individual mice infected with a respiratory bacterium, as measured by photons per unit area (photons s^−1^ cm^−2^) emitted by self-bioluminescent *Bordetella bronchiseptica* (*B. bronchiseptica* lux^+^) from a subsample (*n* = 3) of a larger treatment group of BALB/c mice (*n* = 10) over a 22-day period. The bacterial load is represented by the colours displayed on a rainbow scale, where violet is assigned to the lowest light output and red to the highest, allowing easy identification of bright light regions.

**Figure 3.**
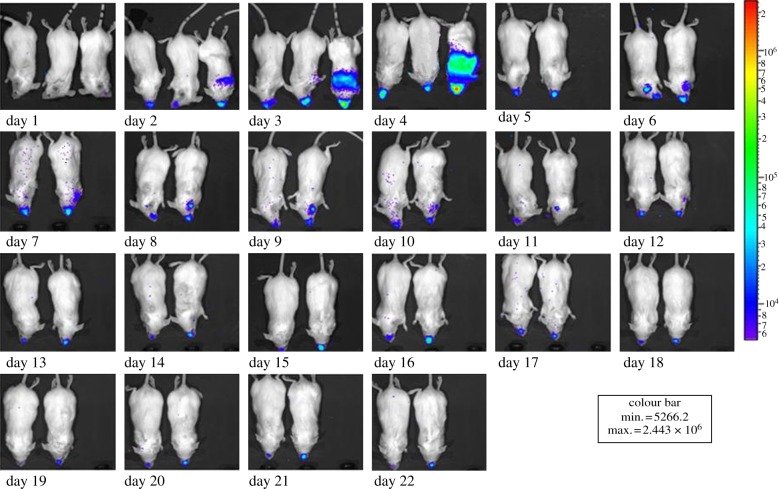
Bacterial load of individual mice over time co-infected with a respiratory bacterium and a gastrointestinal helminth, as measured by photons per unit area (photons s^−1^ cm^−2^) emitted by self-bioluminescent *Bordetella bronchiseptica* (*B. bronchiseptica* lux^+^) from a subsample (*n* = 3) of a larger treatment group of BALB/c mice (*n* = 10) over a 22-day period that were co-infected with the helminth, *Heligmosomoides polygyrus*. The bacterial load is represented by the colours displayed on a rainbow scale, where violet is assigned to the lowest light output and red to the highest, allowing easy identification of bright light regions. Note on day 4 the lux measurement of the animal in position 3 was 2 s.d.s higher than average and it met the criteria for removal from the experiment.

Bacteria were observed in the lungs in some co-infected animals as early as day 2, progressing to the trachea of both single and co-infected animals from day 5 after which a persistent nasal infection occurred in both treatment groups (figures 2 and 3). If bacterial infection reached higher than the average bacterial load by 2 s.d.s (as measured by RLUs), these individuals were assessed to have reached one of the ethical endpoints of the experiment (systemic infection) and according to the guidelines of the IACUC were euthanized (e.g. co-infected individual in position 3 on day 5; figure 3). Taking account of the variance associated with individuals, significantly higher bacterial loads were found in the lungs of co-infected animals compared with single-infected animals (GLMM: *F*_2,28_ = 4.22; *p* = 0.03). There was no significant difference between the mean whole body bacterial load of single and co-infected animals (GLMM: *F*_2,28_ = 2.50; *p* = 0.11) and in the mean bacterial load in the nose and trachea between co-infected and single-infected animals (GLMM: *F*_2,28_ = 1.14; *p* = 0.35).

**Figure 4.**
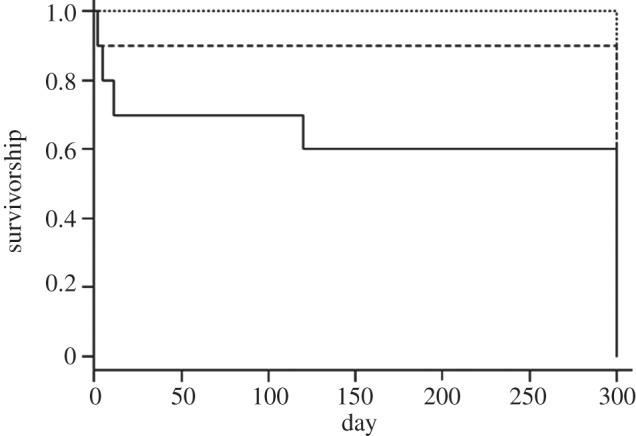
Survivorship curves over 300 days of three different treatment groups (*n* = 10 per groups); infection with the respiratory bacterium *Bordetella bronchiseptica* (dotted line), infection with the gastrointestinal helminth *Heligmosomoides polygyrus* (dashed line) and co-infection with both *B. bronchiseptica* and *H. polygyrus* (solid line).

Using Cox proportional hazards to relate the survivorship to the continuous variable of bacterial loads, we find a significantly higher mortality of co-infected animals over the course of the experiment (*χ*^2^ = 34.2, d.f. = 19, *p* = 0.017; figure 4).

### 3.2. Does co-infection alter helminth egg shedding?

**Figure 5.**
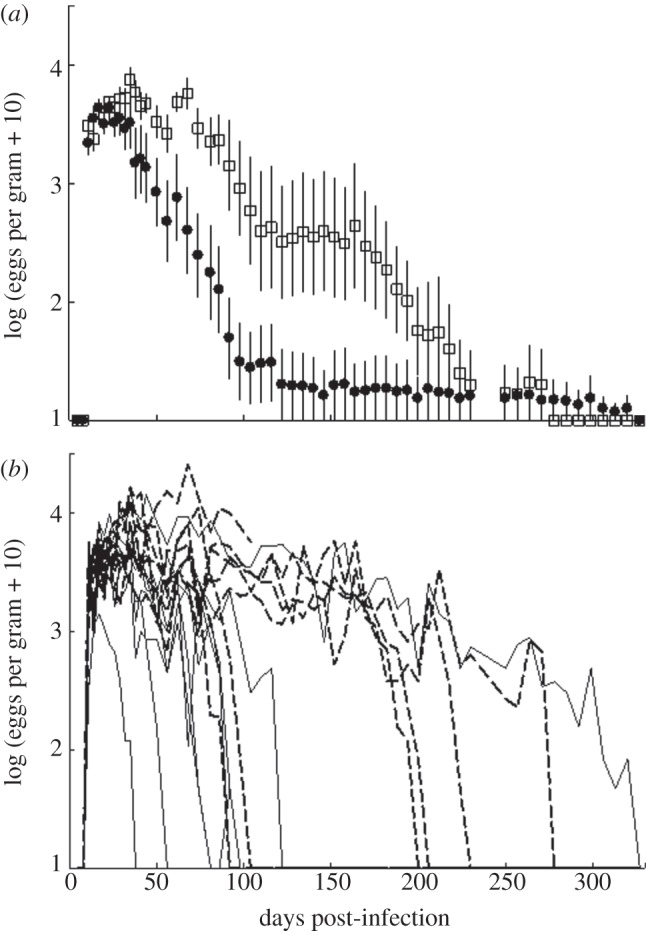
Shedding of helminth eggs in mice infected with a helminth*-*only or co-infected with a bacterium and helminth. (*a*) Mean number of EPG host faeces ± s.e. Black symbols represent hosts infected with the gastrointestinal helminth *Heligmosomoides polygyrus* only, white symbols are hosts simultaneously infected with *H. polygyrus* and the respiratory bacterium *Bordetella bronchiseptica*. (*b*) Number of eggs shed for each host separately. Solid lines represent hosts infected with *H. polygyrus* only, dashed lines are hosts simultaneously infected with *H. polygyrus* and *B. bronchiseptica*.

A cubic smoothing spline was fitted to the course of infection in the random models, which allowed potential nonlinear relationships between single and co-infection growth and establishment to be assessed. Likelihood ratio tests were used to compare the random model (shedding over time) in each analysis. A likelihood ratio test showed the spline was significant (*p* < 0.05). As such, helminth egg shedding was significantly increased in co-infected compared with single-infected mice over the duration of the *in vivo* experiment (*p* < 0.05). The period of egg shedding was not significantly different between single and co-infected hosts (negative binomial GLM: d.f. = 1, *p* = 0.056; figure 5*a*), although this was marginal at the 5 per cent level. One single-infected individual shed helminth eggs for 320 days while all other single-infected individuals shed eggs on average for 79 ± 9 days (mean ± s.e.; figure 5*b*). This individual could be considered a ‘super-shedder’, although it is worth noting that super-shedding was not due to the presence of a secondary infection as these individuals were infected with a helminth-only.

The mean total number of eggs shed over the duration of the experiment (365 days) for the helminth-only infected animals was 1072 ± 498 s.d. To quantify how many super-shedders co-infection generated, we quantified how many individuals from the co-infection treatment group were shedding helminth eggs at a rate 2 s.d.s above single-infected. We determined that five of seven (seven because three had reached such high bacterial loads that they were removed from the experiment) animals in the co-infected groups were super-shedders according to our definition (mean egg shedding of super-shedders = 4120 eggs, over the duration of the experiment).

**Figure 6.**
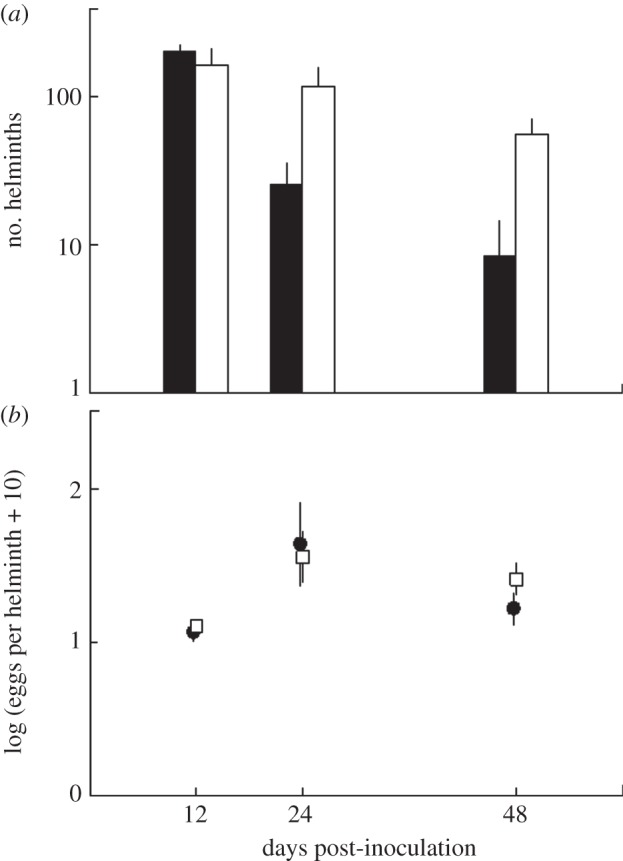
Helminth numbers and *per capita* number of eggs shed from mice that have been infected with either the gastrointestinal helminth *Heligmosomoides polygyrus* alone (black bars/symbols) or co-infected with the helminth and the respiratory bacterium *Bordetella bronchiseptica* (white bars/symbols). Groups of mice were euthanized and dissected 12, 24 or 48 days after infection. (*a*) Numbers of adult *H. polygyrus* in mice infected with the helminth-only or co-infected. (*b*) Number of eggs per adult helminth in single and co-infected mice.

To assess potential differences in clearing of helminths between single and co-infected hosts, the numbers of adult helminths established in the gastrointestinal tract of sacrificed hosts were quantified. Adult *H. polygyrus* were found on days 12, 24 and 48 in both single and co-infected treatment groups. Prior to day 12, adult helminths had not established in either group. Significantly more adult helminths were found in co-infected mice (negative binomial GLM, d.f. = 1, *p* = 0.013; figure 6*a*). The number of helminths in single infections decreased significantly between days 12 and 48, but not in co-infected mice (negative binomial GLM, time: d.f. = 1, *p* < 0.001, figure 6*a*). There was a marginally significant interaction between time and infection status with a stronger decrease of helminths in single compared to co-infected hosts (negative binomial GLM, time: treatment: d.f. = 1, *p* = 0.053). As we were interested in the consequences of co-infection on transmission potential, we calculated the number of eggs shed by each helminth, using eggs collected from host faeces prior to destructive sampling (figure 6*b*). Single and co-infected individuals, however, did not differ in the *per capita* (per helminth) number of eggs (negative binomial GLM, treatment: d.f. = 1, *p* = 0.873).

To determine whether helminth eggs shed from single and co-infected mice differed in their viability (i.e. their development to larvae) and to assess the relationship between the number of eggs shed and development to infectious L3 larvae, i.e. whether egg shedding is a good proxy for infectiousness, we compared their development with the infectious larval stage. On day 21 post-inoculation, the numbers of eggs shed were 12.7 ± 2.6 s.e. EPG faeces in single infected and 14.2 ± 3.5 s.e. EPG of faeces in co-infected hosts. No significant difference was found in the viability of the L3 larvae between single and co-infected animals (log mean number of hatchlings ± standard error: single = 4.57 ± 0.05; co-infected = 4.74 ± 0.12; GLM *F*_1,14_ = 1.765, *p* = 0.205).

## 4. Discussion

Co-infected individuals shed significantly more helminth eggs for an extended period of time, and bacterial load was significantly higher in the lungs of co-infected rather than single-infected individuals. Co-infection created helminth super-shedders, those individuals that shed significantly higher number of eggs than average, over the duration of the experiment. While helminth egg shedding can be related to infectiousness, relating the bacterial load *in vivo* to host infectiousness is, however, more complicated. Shedding of *B. bronchiseptica* has been shown to be positively affected by the number of bacteria in rabbit hosts [39]. *Bordetella bronchiseptica* is a natural parasite in mice [40,41], and transmission between female mice and their offspring has been observed in transmission experiments under controlled laboratory conditions (S. Lass 2008, unpublished data), as such, a high bacterial load may translate into higher infectiousness, although further empirical work is required for validation. Thus, we show that co-infection could be a factor contributing to the commonly observed variation in both individual infection load and host infectiousness and may therefore alter the dynamics of epidemics [2–7].

Co-infection is not without a cost to the host. In our experiment, co-infected individuals suffered from higher mortality than individuals infected with the bacteria or helminth only (figures 3 and 4). We observed the proliferation of one parasite (bacteria) to be elevated due to the presence of a secondary infection (helminth), which led to increased parasite-induced host mortality. This has been shown to occur at a population level, whereby co-infecting parasites caused ecological interference thereby strongly affecting parasite dynamics [42]. Given that helminth egg shedding is a good proxy of infectiousness, if co-infections increase the heterogeneity in infection intensities then co-infected individuals may act to increase the basic reproduction number (*R*_0_) of a parasite. Alternatively, the cost associated with co-infection could be such that those individuals have little overall contribution to *R*_0_ because they are rapidly removed from the epidemic owing to co-infection-induced host mortality. In addition, of the individuals that were co-infected five of seven developed into helminth super-shedders and so the co-infection-induced mortality may act to reduce population-level transmission potential of the helminths. To determine the role of individual hosts in the dynamics of infectious disease, however, we need to know simultaneously the number of contacts and the infectiousness of that individual; data not collected here. To the best of our knowledge, host infectiousness and contact frequency have not been examined simultaneously. However, it has been found that cattle co-penned with super-shedders had significantly greater mean pen *E. coli* levels than animals that were not co-penned animals [43].

Interestingly, the variance in bacterial loads and egg shedding within treatment groups was high. One helminth-only infected host shed parasite eggs for 320 days while all other single-infected individuals had cleared helminth infection on average by day 79±9 days post-infection (figure 5). This single-infected individual meets our criteria for definition as a super-shedder although mechanisms other than co-infection have generated this extended shedding. In addition, bacterial load in some, but not all co-infected hosts reached higher than average loads by 2 s.d.s (figure 3). Given the homogeneous environmental conditions in the laboratory and the low genetic diversity of the BALB/c mouse strain and the parasites used, this variation within the co-infected and helminth-only treatment group was surprising, but similar patterns have been observed in host–parasite interactions with clonal hosts [44,45]. Exposing different host clones to different doses of parasite isolates in a well-controlled laboratory experiment resulted in considerable variation in infection probability [45]. Non-inherited phenotypic differences such as differences in the immune response or in life-history traits may be the underlying cause for the observed variation among individual hosts [45]. This could also be true for our experiment. The observed variation in shedding helminth eggs and bacterial load may be caused by external factors (e.g. micro-environmental variation between our mouse cages) or by internal factors such as molecular mechanisms of the immune system (e.g. alternative splicing [46]) or within-mouse strain variation, e.g. in life-history traits. Indeed, our observations show that while co-infection certainly seems to generate super-shedders it is not the case that all super-shedders are co-infected.

Our experiment showed that helminth establishment was initially the same in single and co-infected groups (figure 6*a*), but clearance was faster in single-infected individuals. Thus, the difference in helminth infection intensity in co-infection versus single infection was likely to be mediated by the host's immune system. While parasites in the same location within a host may interact due to competition for resources, including space and nutrients, interactions between parasites in different physical locations are likely to be mediated by the immune system [13,19], and these interactions may be antagonistic towards parasite defence [13,47–49]. Previous work has shown that clearance of *B. bronchiseptica* from the lungs requires IFN-γ, part of a Th1-mediated response [21], whereas an immune response to *H. polygyrus* is initiated by Th2 cytokines, such as IL-4 and IL-10 in the murine host [50]. The anticipated antagonism between these immune responses to the bacterial–helminth co-infection may therefore have produced the impaired clearance of *B. bronchiseptica* and *H. polygyrus* [13,20]. Alternatively, the immunomodulatory pathways known to be activated by *H. polygyrus* [51–53] could lead to suppression of immune responses to both parasites, thereby producing the helminth super-shedders and the high bacterial loads observed.

Bioluminescent imaging (BLI) has previously been used to study non-infectious disease progression and infectious disease colonization processes [53], but the application in disease ecology and the investigation of co-infection used here was novel. Using real-time, whole animal, *in vivo* monitoring of *B. bronchiseptica* infection allowed us to determine spatial location, real-time infection load, observe high bacterial loads early in the infection (day 2) and to significantly reduce the number of animals used in the experiment, thereby contributing to the 3Rs for animal use in research (reduction, refinement and replacement). It is worth noting that if we were to have applied a ‘traditional regime’ of sacrificing animals to determine bacterial infection load this same experiment would have required 264 animals, as opposed to the 40 used (an 85% reduction in animal use).

Imaging bacterial infection models with the use of a bacterial lux operon allows for real-time monitoring and quantification of infection, without requiring administration of a substrate; an obviously useful tool for infectious disease ecology. BLI is in its infancy in infectious disease research, but where it has been used novel observations have occurred, mostly due to the increased temporal and spatial resolution available on the infections due to real-time and *in vivo* observations, including novel sites of pathogen replication [54,55]. Here, we have observed high infection loads that may have been missed if the traditional techniques of observing infection every 2–7 days had been used [21,56].

Furthermore, traditional infection models have required host sacrifice to quantify bacterial load from individual host organs during infection. This typically involves sacrificing at least three mice at set time points during an experiment. As such, daily measurements of infection load are often not ethically possible owing to the large number of mice that would be required to run such an experiment. BLI, however, provides a unique opportunity to longitudinally monitor infection in a single host over a fine temporal scale (in our case daily). In addition, because imaging occurs *in vivo* and light is emitted when the bacteria are metabolically active the counts observed may be a better reflection of actual bacterial numbers than other *in vivo* techniques such as gfp-reporters and traditional techniques such as counting CFUs on growth media.
